# Accidental apixaban intoxication in a 23-month-old child: a case report

**DOI:** 10.1186/s12887-020-02448-4

**Published:** 2020-12-05

**Authors:** Manon Launay, Yara Nasser, Isabelle Maubert, Anne-Cécile Chaux, Xavier Delavenne

**Affiliations:** 1 Laboratoire de Pharmacologie-Toxicologie-Gaz du Sang, CHU Saint-Etienne, Albert Raimond avenue, Saint-Etienne, France; 2 Laboratoire d’analyses médicales, CH Emile Roux, le Puy-en-Velay, France; 3 Service de Médecine Intensive et Réanimation Pédiatrique, CHU Saint-Etienne, Saint-Etienne, France; 4 INSERM U1059, Dysfonctions Vasculaires et de l’Hémostase, Université de Lyon, Saint-Etienne, France

**Keywords:** Apixaban, DOAC, Pediatrics, Intoxication, Pharmacokinetics, Child, Overdose, Digoxin, Case report

## Abstract

**Background:**

Direct oral anticoagulants, such as apixaban, are increasingly used in everyday practice in order to treat or prevent thromboembolic diseases. To date, there is no available data about apixaban pharmacokinetics in children, and no intoxication has previously been described.

**Case presentation:**

A 23-month-old boy, with no medical history, was admitted to the emergency department 2 h after accidentally ingesting 40 mg apixaban and 0.75 mg digoxin. No adverse event was observed. Digoxin trough level was within therapeutic values. Apixaban blood concentration increased up to 1712 μg/L at H + 6 (1000–2750 μg/L using 2–5 mg/kg of apixaban in adults). The terminal half-life was 8.2 h (6–15 h in adults). The rapid elimination may explain the absence of bleeding despite high concentrations.

**Conclusions:**

Despite an important intake of apixaban and a real disturbance in routine coagulation assays, no clinical sign of bleeding was observed, perhaps due to wide therapeutic range of apixaban. It may also be explained by its rapid elimination. Considering the high Cmax and a possible enteroenteric recycling, the use of activated charcoal should be considered in such situations in order to prevent eventual bleeding.

## Background

Direct oral anticoagulants, such as apixaban, are increasingly used in everyday practice in order to treat or prevent thromboembolic diseases [1, 2]. Apixaban is a reversible and selective FXa inhibitor (activated factor X) and inhibits free, clot-bound FXa, and prothrombinase activity [3]. Apixaban has linear pharmacokinetics, and concentration-related pharmacodynamic effects have been described in adults [4]. Many clinical cases reported severe self-poisoning with apixaban among adult patients [5–9]. To date, there is no available data about apixaban pharmacokinetics in children, and no intoxication has previously been described. Digoxin, on the other hand, is better known in pediatrics as it is commonly used for arrhythmias and heart failure treatment.

## Case presentation

**Fig. 1:**
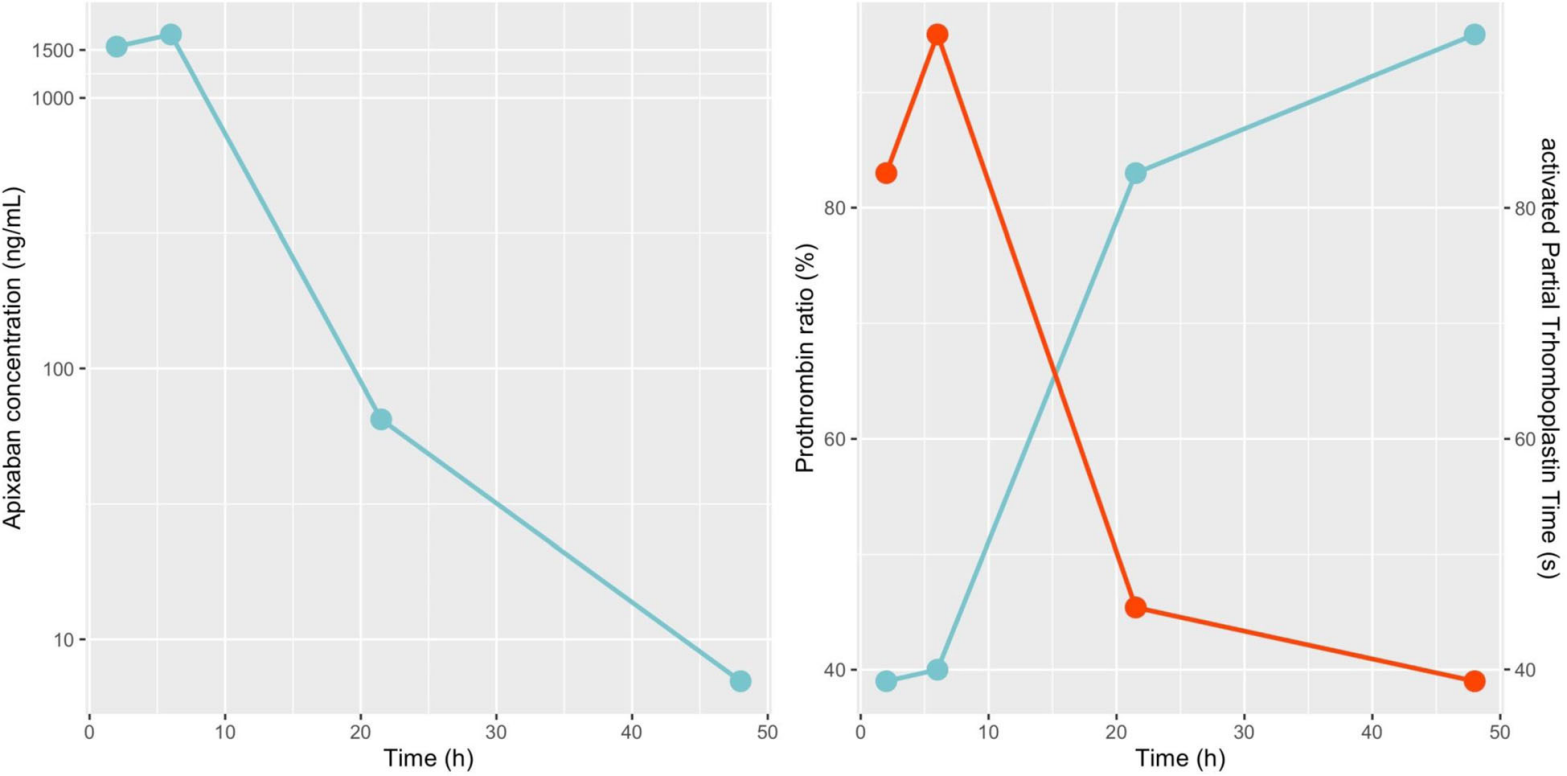
Variation of apixaban (μg/L), PT (%) (blue line) and aPTT ratio (red line) of a 23-month-old child at different times after accidentally ingesting 40 mg of apixaban and 0.75 mg of digoxin

A 23-month-old boy (12.9 Kg), with no medical history, was admitted to the emergency department 2 h after accidentally ingesting 8 pills of apixaban 5 mg (40 mg) and 3 pills of digoxin 0.25 mg (0.75 mg). His clinical exam was normal. No hemorrhagic sign was identified. His heart rate (105 pulsations/minute) and the ECG were normal. The child remained under medical supervision for 48 h. Four blood tests were withdrawn during hospitalization at 2, 6, 21.5 and 48 h after the ingestion (H + 2, H + 6, H + 21.5, H + 48), for digoxin concentrations monitoring and routine coagulation assays. After H + 6, the child was transferred to the University Hospital of Saint Etienne, where apixaban monitoring was available using a published Liquid Chromatography-Mass Spectrometry method [10]. Apixaban monitoring was initiated at H + 21.5. Apixaban concentrations were also retrospectively analyzed at H + 2 and H + 6. The renal function was normal and remained stable (Creatinine between 17 and 24 μmol/L (normal range: 15–35 μmol/L [11])). No clinical sign of bleeding was observed. Apixaban concentration increased up to 1712 μg/L at H + 6, then decreased to 7 μg/L at H + 48 (Fig. 1). Apixaban was eliminated with a terminal half-life of 8.2 h and its distribution volume indexed to bioavailability was 23 L or 1.8 L/Kg. As expected, aPTT ratio (activated partial thromboplastin time ratio: Patient aPTT (sec) /Normal plasma aPTT (sec)) and PT were prolonged [4] (Fig. 1).

Regarding digoxin, the concentration at H + 2 was 5.9 μg/L and decreased to 0.4 μg/L at H + 48. Digoxin was eliminated with a half-life of approximatively 15.6 h.

The patient left the hospital after 48 h without any complication or sequelae.

## Discussion/conclusion

To our knowledge, this is the first case reporting apixaban intoxication in children. Apixaban blood concentration increased up to a high level of 1712 μg/L at H + 6 after ingesting 40 mg of apixaban (3.1 mg/Kg). Cmax was consistent with data in adults overdose (between 1000 and 2750 μg/L after ingesting 2 to 5 mg/kg of apixaban) [5, 6].

Despite multiple ongoing studies on apixaban in the pediatric population [12–15], there is currently no available data on apixaban pharmacokinetics and pharmacodynamics properties among children. As previously reported, the apixaban plasma concentration vs. time profile exhibited a multiphasic elimination profile, with an initial rapid decline followed by a more gradual terminal phase [4]. Apixaban was eliminated with a terminal half-life of 8.2 h. The half-life described in adults overdose was between 6 h and 15 h depending on the patient and other co-administered drugs [6–9]. The rapid elimination may explain the absence of bleeding despite high concentrations. The results observed during the clinical development of new oral anticoagulants have shown that the inexplicable variability of drug response is quite low in highly selected populations, so there is no sense in recommending drug monitoring for such patients. However, sources of inter- and intra-individual variability (such as renal and/or hepatic function, advanced age, relevant drug-drug interaction, …) have been identified, concerning a restricted population at very high risk of clinical events [16]. Drug overdose could also be at high risk of clinical event and the monitoring of apixaban concentrations should be assessed for these patients, if available. As no apixaban dosage was performed within the first 20 h after the ingestion, the patient was followed-up with PT and aPTT ratio monitoring. As expected, because apixaban is a reversible anti-Xa with concentration-related pharmacodynamic effects, aPTT ratio and PT were prolonged with an interesting overlap as shown in Fig. 1. Thus, routine coagulation assays could be monitored when apixaban monitoring is not available. The distribution volume indexed to bioavailability was markedly different than the one reported in adults, 23 L vs 73 L in adults, or 1.8 L/Kg vs 0.9 L/Kg [4].

Furthermore, a peak concentration was probably reached between H + 2 and H + 6 and apixaban was still detectable at H + 48 (Fig. 1), with a possible enteroenteric recycling (i.e., reabsorption of drug excreted from the systemic circulation directly into the intestine) [17]. The use of activated charcoal in similar cases would be interesting in order to prevent eventual bleeding. A 28% decrease in apixaban exposure was indeed observed following administration of activated charcoal at 6 h post-dose [17]. Apixaban monitoring was done retrospectively in this case, and no antidote was administered but no side effect was reported.

The patient has also ingested 0.75 mg (0.06 mg/kg) of digoxin when the mean therapeutic dose is around 0.01 mg/kg/day for children [18–20]. The half-life was 15.6 h, a figure markedly shorter than the described half-life in the pediatric population (36 h) [18, 19]. Digoxin pharmacokinetic parameters have indeed already shown extensive variation in children, with clearance ranging from 6.0 to 3331.8 mL/kg/h [21]. Digoxin level at 24 h was 1.3 μg/L, which is an usual therapeutic value [18–20, 22], and no adverse event was observed. Toxicity (such as nausea, vomiting and ECG abnormalities, etc.) is indeed frequently reported for trough level greater than 2 μg/L [20, 22].

Despite an important intake of apixaban and a real disturbance in routine coagulation assays, no clinical sign of bleeding was observed, perhaps due to wide therapeutic range of apixaban. It may also be explained by its rapid elimination. Considering the high Cmax and a possible enteroenteric recycling, the use of activated charcoal should be considered in such situations in order to prevent eventual bleeding.

## Data Availability

The data that support the findings of this study are available from the corresponding author upon reasonable request.

## Abbreviations

aPTT: activated partial thromboplastin time
FXa: activated factor X
